# Whole almond consumption is associated with better diet quality and cardiovascular disease risk factors in the UK adult population: National Diet and Nutrition Survey (NDNS) 2008–2017

**DOI:** 10.1007/s00394-020-02270-9

**Published:** 2020-05-16

**Authors:** Vita Dikariyanto, Sarah E. Berry, Lucy Francis, Leanne Smith, Wendy L. Hall

**Affiliations:** Diet and Cardiometabolic Health Research Group, Department of Nutritional Sciences, Faculty of Life Sciences and Medicine, King’s College London, London, SE1 9NH UK

**Keywords:** Almonds, Cross-sectional analysis, Diet quality, Cardiovascular disease, Nutrients

## Abstract

**Purpose:**

This work aimed to estimate whole almond consumption in a nationally representative UK survey population and examine associations with diet quality and cardiovascular disease (CVD) risk.

**Methods:**

Four-day food record data from the National Diet and Nutrition Survey (NDNS) 2008–2017 (*n* = 6802, age ≥ 19 year) were analyzed to investigate associations between whole almond consumption and diet quality, measured by the modified Mediterranean Diet Score (MDS) and modified Healthy Diet Score (HDS), and CVD risk markers, using survey-adjusted multivariable linear regression.

**Results:**

Whole almond consumption was reported in 7.6% of the population. Median intake in whole almond consumers was 5.0 g/day (IQR 9.3). Consumers had higher diet quality scores relative to non-consumers; higher intakes of protein, total fat, monounsaturated, *n-*3 and *n*-*6* polyunsaturated fats, fiber, folate, vitamin C, vitamin E, potassium, magnesium, phosphorus, and iron; and lower intakes of *trans*-fatty acids, total carbohydrate, sugar, and sodium. BMI and WC were lower in whole almond consumers compared to non-consumers: 25.5 kg/m^2^ (95% CI 24.9, 26.2) vs 26.3 kg/m^2^ (25.9, 26.7), and 88.0 cm (86.2, 89.8) vs 90.1 cm (89.1, 91.2), respectively. However, there were no dose-related fully adjusted significant associations between increasing almond intake (g per 1000 kcal energy intake) and lower CVD risk markers.

**Conclusions:**

Almond intake is low in the UK population, but consumption was associated with better dietary quality and lower CVD risk factors. Habitual consumption of whole almonds should be encouraged as part of a healthy diet.

**Electronic supplementary material:**

The online version of this article (10.1007/s00394-020-02270-9) contains supplementary material, which is available to authorized users.

## Introduction

Urgent calls for a revolution in global food systems have been made to meet the United Nations (UN) Sustainable Development Goals (SDGs) and Paris Agreement to eradicate malnutrition and non-communicable diseases (NCDs) while conserving the environment and biodiversity [1–4]. The EAT Lancet report set dietary targets for healthy diets from sustainable food systems, including a doubling of consumption of fruits, vegetables, legumes and nuts. Almonds are the most commonly consumed tree nut in many countries, with global agricultural production of 2018/2019 having increased by 20% compared to a decade ago [5]. North America accounted for the world’s highest production of tree nuts, but Europe was the largest consumer worldwide. Hence, many of the main importing countries were in Europe, including Spain, Germany, Italy, France, the Netherlands and the UK [5].

Almonds are characterized as nutrient-dense foods, being rich in protein, unsaturated fatty acids, dietary fiber, and micronutrients [6, 7], as well as having a low glycemic load attribute which have been linked to lower cardiometabolic disease risk [8–10]. Almonds are also a source of beneficial non-nutrient bioactives, such as (poly)phenolic compounds [11–13]. According to a qualified health claim issued by US Food and Drug Administration (FDA) in 2003, “scientific evidence suggests but does not prove that eating 1.5 oz (42.5 g) per day of most nuts, such as almonds, as part of a diet low in saturated fats and cholesterol, may reduce the risk of heart disease” [14]. Randomized controlled trials (RCTs) have provided evidence that almond consumption lowers blood LDL-cholesterol and maintains or increases HDL-cholesterol concentrations, lowers blood glucose levels, as well as some inflammatory markers [15–23]. Regarding weight management, high doses of almonds incorporated in a diet have been shown to cause a greater reduction of weight/body mass index (BMI), waist circumference (WC) and fat mass in overweight and obese subjects in comparison with a complex carbohydrate-enriched diet [24], although doses of < 42.5 g/day were not effective for weight loss in a meta-analysis [23].

Almonds can be consumed whole, chopped, sliced, ground, roasted, raw, blanched, salted, coated with chocolate or sweetened, or as an oil, butter or paste. Whole kernels, a convenient snack food, are the most efficient way of consuming quantities sufficient to modify LDL-cholesterol concentrations. Very little is known about population-level intakes of almonds. An observational study in USA adults (≥ 19 year) using data from the National Health and Nutrition Examination Survey (NHANES) 2001–2010 (*n* = 24,808) revealed that the prevalence of almond consumption (including whole almond kernels, with and without salt, almond butter, and almond paste) measured by 24 h dietary recalls was 1.6% [25]. This study also revealed that almond consumption (estimated usual intake 29.5 g/day) was associated with lower BMI and WC, and that consumers had better diet quality and greater nutrient adequacy than non-consumers [25]. Therefore, the current study aimed to investigate associations between whole almonds and diet quality, nutrient intakes, as well as CVD risk markers using 4 day food records from a nationally representative population of 6802 adults who participated in the UK National Diet and Nutrition Survey (NDNS) rolling program 2008–2017 [26]. It was hypothesized that whole almond consumption was linked to higher diet quality scores, better nutrient intakes, and improved profile of intermediary CVD risk factors.

## Materials and methods

### The National Diet and Nutrition Survey Rolling Programme (NDNS-RP) and study population

The NDNS-RP is a long-running government-funded scheme to assess diet, nutrient intake and nutritional status of the general population (> 1.5 year) living in private households in the UK (England, Scotland, Wales and North Ireland) [26–29]; the study is registered with the ISRTCN registry as ISRCTN17261407. Random sampling was carried out on addresses throughout the UK listed in Postcode Address File (PAF). Of all the addresses, Primary Sampling Units (PSUs) were created to make small clusters of geographical area based on postcode sectors to increase cost effectiveness. The randomly selected addresses were drawn from each PSU. An adult in each household was randomly selected, and where a single address had multiple households, a household was also selected randomly. Full details on the random selection procedure are available at the NDNS User Guide [30].

The cross-sectional analysis reported here included data from adult participants (≥ 19 year, *n* = 6802), who completed at least 3 days of 4 days estimated food diary in the NDNS-RP 2008–2017 (Year 1–9) [26–29]. Of 6802 adult respondents, 147 individuals completed only 3 days of 4 days estimated food diary and the remainder of the sample completed all 4 days. Participants were asked to record all food and drink consumed over 4 consecutive days comprising 3 week-days and a weekend day, including portion sizes, brand names, and recipes for home cooked foods. Food and drink items were assigned a code and dietary analysis was conducted using the DINO (Diet in Nutrients Out) platform based on Public Health England’s NDNS Nutrient Databank food composition data.

### Ethics

For NDNS RP 2008–2013, ethical approval was obtained from the Oxfordshire A Research Ethics Committee (Ref. No. 07/H0604/113) and for NDNS RP 2014–2017, the approval was received from the Cambridge South NRES Committee (Ref. No. 13/EE/0016) [31]. Informed consent was obtained from every participant. The survey involved interview visits for questionnaires, 4 days food diaries, and a nurse visit for anthropometry and physical measurements and also blood and 24 h urine sample collections [26–29].

### Definition of almond consumption

The intake of raw and roasted whole almonds was defined and determined both as a single nut product (almond kernel only), and also total almond kernel intake where also derived from mixed nut/fruit and nut products. Thus, whole almond consumption was defined as: (1) any amount of intake of whole almond kernels only (AKO), or (2) AKO in addition to any amount of intake of almond kernels from mixed nut products and mixed nut and fruit products (AKM). Data related to almond consumption were isolated from the NDNS Year 1–9 database, i.e. ALMONDS KERNEL ONLY, MIXED NUTS AND RAISINS UNSALTED, MIXED NUTS KERNELS ONLY SALTED, MIXED NUTS UNROASTED UNSALTED and TRAIL HAWAIIAN TROPICAL MIX MIXED NUTS DRIED FRUIT. It was necessary to estimate the amount of whole almond kernels in mixed nut products and mixed nut and fruit products by market sampling. Mixed nut products containing almond kernels from 19 brands were purchased from UK supermarkets, such as Tesco, Sainsbury’s, Waitrose, M&S, ASDA, Coop and Lidl. Almonds contained in these mixed nut/fruit and nut products were weighed manually and the percentage of almond kernel portion in comparison with the total weight of the products was calculated in order to estimate total intakes of whole almond kernels from both mixed nut/fruit and nut products and almond kernel only products (see Supplementary material).

### Diet quality indices

To estimate diet quality, two existing diet scores were adapted for the current study: the Mediterranean Diet Score (MDS) [32] and Healthy Diet Score (HDS) [33]. Maynard et al*.* (2004) developed the HDS based on Healthy Diet Indicator (HDI) and the UK guidelines at that point in time, as recommended by the Committee on Medical Aspects of Food Policy (COMA) [33]. Modifications were applied to HDS for this study to reflect UK current recommendations [27, 34–38], and nuts were removed from the MDS scoring system as appropriate for this study on diet and health associations with nut consumption. The potential top score of the modified MDS remained the same: 9, but the modified HDS had a potential top score of 14, while the original HDS scoring range was 0–12. Tables A1 and A2 in supplements show original and modified items of MDS and HDS items, respectively.

### Cardiovascular disease risk markers

Body mass index (BMI; kg/m^2^), waist circumference (WC; cm), systolic blood pressure (SBP; mmHg), diastolic blood pressure (DBP; mmHg), total cholesterol (TC; mmol/l), triglycerides (TAG; mmol/l), high-density lipoprotein (HDL-C; mmol/l), low-density lipoprotein (LDL-C; mmol/l), TC:HDL-C (the ratio of TC and HDL-C) and C-reactive protein (CRP; mg/l) were the CVD risk markers included in the analysis. Interviewer measurement protocols and procedures for blood sample collection, processing, analysis and quality controls are detailed elsewhere [26–29]. Body height and weight were measured using a portable stadiometer and a weight scale, and BMI was calculated by fieldworkers. Waist circumference measurement was taken using a tape measure. The discrepancy tolerances of repeat measurement readings were not detailed in the NDNS method protocols. Omron HEM907, an automated validated monitor, was used to measure blood pressure in a sitting position after a 5-min rest. Trained fieldworkers took blood pressure measurements three times and results were presented based on the mean value of second and third readings with one-minute intervals [26–29].

### Statistical analysis

Statistical analysis was carried out using SPSS IBM 23 and a two-sided *P *value of 0.05 was considered statistically significant. Data are presented as adjusted means (95% CI) for individual nutrient intakes, total diet quality scores as well as levels of CVD risk markers, and as medians (with IQRs) for amount of whole almonds consumed and age. To examine whether there was a statistically significant association between almond consumption and alcohol and total energy intakes as well as demographic variables, i.e. age, sex, ethnicity, socio-economic and smoking status and region of residency, survey-adjusted generalized linear model (GLM) with a binary logistic link function was used. Survey-adjusted GLM with a linear link function (predictors: age, sex, ethnicity, socio-economic and smoking status, region of residency, total energy and alcohol intake) was used to examine whether there were significant differences between whole almond consumers and non-consumers in their diet quality scores, nutrient intakes and CVD risk markers. These predictors were included due to their associations with CVD to determine whether differences in consumer groups were independent of these factors. Age, sex and ethnicity are known influencing factors in CVD risk development [39]. Socio-economic status has been reported to be associated with CVD risk [40] and may influence purchasing capacity for food. Smoking has proatherogenic effects via vascular dysfunction [41]. Energy and alcohol intake are dietary determinants of CVD; excess calorie is associated with obesity which is included in the pathophysiological pathway of CVD [42, 43]. Region of residency is considered to have influences on market access for almond and mixed nut or mixed nut and fruit products which further affect consumer access.

To investigate dose–response associations between whole almond consumption (g/1000 kcal energy intake) and diet quality and CVD risk markers, survey-adjusted multivariable linear regression models were used adjusting for the same covariates mentioned above. Normal residual distributions were checked by visual inspection of histograms and Q–Q plots; data with non-normally distributed residuals were log transformed using log_10_ for analysis of survey-adjusted GLM and multivariable linear regression. The results of analysis were back transformed into the geometric mean values. Homoscedasticity was checked by plotting the standardised residuals of dependent variables and predictors.

During the analysis, the weight factor provided by the NDNS database resource was applied to adjust for non-response and known socio-economic differences in the survey to ensure that the data were nationally representative for the UK population and reducing selection bias and non-response bias [30, 44]. The weight factor used was wti_Y19 (Weight for individual and diary-all ages, combined Year 1–9 (the UK NDNS-RP 2008–2017)) for investigating differences in diet quality scores and nutrient intakes between whole almond consumers and non-consumers, associations between almond consumption and demographic variables, and multivariable linear regression including diet quality scores. Weight factors wtn_Y19 (Weight for nurse-all ages, combined Year 1–9 (the UK NDNS-RP 2008–2017)) was used for GLM and multivariable linear regression including variables BMI, waist circumference and blood pressure; and wtb_Y19 (Weight for blood-all ages, combined Year 1–9 (the UK NDNS-RP 2008–2017)) was used for GLM and multivariable linear regression for blood analyte variables including C-reactive protein and lipids [30, 44].

## Results

### Sociodemographic and lifestyle characteristics

Mean and median intakes in the total study population (consumers and non-consumers combined) were 9.2 g/day (SD 12.4 g/day) and 5.0 g/day (IQR 9.3 g/day), respectively, ranging from < 0.01 to 109.9 g/day. Table 1 shows background characteristics of almond consumers and non-consumers. Median AKO (almond kernels only, *n* = 317, 4.7% of total adult population) and AKM (almond kernels plus almond kernels in mixed nut products and mixed nut and fruit products, *n* = 481, 7.1% of total adult population) consumption contributed 1.1% and 1.7% of total energy intake respectively. On average whole almond consumers were significantly 2 years older than non-consumers and were more likely to be female and non-smokers. A greater proportion of whole almond consumers identified as non-white and reported having lower or high managerial and professional occupations. Furthermore, a greater proportion of AKM consumers resided in England compared to non-consumers.

**Table 1:** Background characteristics of whole almond consumers compared to non-consumers in the UK adult population (≥ 19 year) based on NDNS 2008–2017

	Total adult population	AKO			AKM		
		Consumer, *n* = 317	Non-consumer, *n* = 6,485	*P* value	Consumer, *n* = 481	Non-consumer, *n* = 6,321	*P* value
Amount of almonds consumed (Median (IQR))							
Gram		3.0 (5.9)			5.0 (9.3)		
% total energy intake		1.1 (1.9)			1.7 (3.1)		
Age (median (IQR))	49 (27)	50 (24)	49 (28)	0.298	51 (24)	49 (28)	0.001*
Sex							
Male (%)	41.3	28.7	41.9	< 0.001*	32.5	41.9	< 0.001*
Female (%)	58.7	71.3	58.1		67.5	58.1	
Ethnicity							
White (%)	92.7	86.4	93.0	< 0.001*	88.1	93.0	< 0.001*
Mixed ethnic group (%)	0.9	0.9	0.8		1.0	0.8	
Black or Black British (%)	2.1	1.9	2.1		1.7	2.1	
Asian or Asian British (%)	3.1	8.8	2.8		7.1	2.8	
Any other group (%)	1.2	1.9	1.2		2.1	1.2	
Region							
England (%)	57.4	68.1	56.9	0.130	65.5	56.9	0.033*
Scotland (%)	15.8	8.2	16.1		8.7	16.1	
Wales (%)	14.0	15.1	13.9		14.8	13.9	
Northern Ireland (%)	12.8	8.5	13.0		11.0	13.0	
Socio-economic status							
Higher managerial and professional occupations (%)	15.2	26.4	14.7	< 0.001*	26.8	14.7	< 0.001*
Lower managerial and professional occupations (%)	24.1	31.2	23.7		29.5	23.7	
Intermediate occupations (%)	9.9	9.2	10.0		10.3	10..0	
Small employers and own account workers (%)	10.7	12.4	10.6		12.1	10.6	
Lower supervisory and technical occupations (%)	9.3	7.3	9.4		6.7	9.4	
Semi-routine occupations (%)	14.3	7.6	14.7		8.6	14.7	
Routine occupations (%)	12.1	2.9	12.5		3.6	12.5	
Never worked (%)	2.9	1.9	3.0		1.3	3.0	
Other (%)	1.4	1.0	1.4		1.3	1.4	
Smoking status							
Current smoker (%)	22.6	8.8	23.3	< 0.001*	10.2	23.3	< 0.001*
Ex-Regular smoker (%)	24.3	25.9	24.2		24.9	24.2	
Never regular smoker (%)	53.1	65.3	52.5		64.9	52.5	
Alcohol intake (g/day) (median (IQR))	0.8 (16.4)	4.9 (16.2)	0.5 (16.4)	0.078	4.9 (16.2)	0.5 (16.4)	0.092
Energy intake (kcal/day) (unadjusted mean ± SD))	1760.1 ± 564.7	1876.3 ± 520.8	1754.4 ± 566.2	< 0.001*	1876.3 ± 520.8	1754.4 ± 566.2	< 0.001*

This is a descriptive table. Survey-adjusted GLM with a linear binary logistic function was used to investigate the association between whole almond consumption and demographic variables

*AKO* almond kernel only, *AKM* almond kernel only plus almond kernel in mixed nuts

*P was < 0.05 indicating a significant association, *n* = 6,802

### Diet quality scores

Modified MDS and modified HDS were significantly higher (*P* < 0.001) in AKO consumers (estimated marginal mean modified MDS 5.5; 95% CI 5.3, 5.7; estimated marginal mean modified HDS 6.4; 95% CI 6.2, 6.6) compared with non-consumers (estimated marginal mean MDS 4.7; 95% CI 4.6, 4.8; estimated marginal mean modified HDS 5.7; 95% CI 5.6, 5.8). Results for AKM consumers were almost identical (data not shown).

### Nutrient intake

Almond consumers had significantly higher total energy and food energy intake (10% higher), as well as greater intakes of fat, cis-monounsaturated fatty acids, cis n-6 fatty acids, cis n-3 fatty acids, intrinsic milk sugars, and fiber intakes, as shown in Table 2. Trans-fatty acids, total carbohydrate, starch, non-milk extrinsic sugars, intrinsic milk sugar and starch intakes were significantly lower in consumers. For micronutrients, as shown in Table 2, fully adjusted analysis revealed that almond consumers, relative to non-consumers, had significantly higher intakes of vitamin E, thiamin, riboflavin, folate, pantothenic acid, biotin, vitamin C, potassium, magnesium, phosphorus, iron, copper, zinc, manganese and selenium, and lower intakes of sodium and chloride. However, there were no differences between groups for vitamin A, vitamins D, riboflavin (AKO only), niacin equivalents, vitamin B12, calcium and iodine. Vitamin B6 was observed to be lower in only AKO consumers compared to non-consumers.

**Table 2:** Daily energy, macro- and micronutrient intake of whole almond consumers and non-consumers, in the UK adult population (≥ 19 year) based on NDNS 2008–2017

Macronutrient (diet only, % food energy)^a^	Estimated marginal mean (95% CI)					
	AKO			AKM		
	Consumers, *n* = 317 (4.7% of total adult population)	Non-consumers, *n* = 6485 (95.3% of total adult population)	*P* value	Consumers, *n* = 481 (7.1% of total adult population)	Non-consumers, *n* = 6321 (92.9% of total adult population)	*P* value
Total energy (kcal)	1851 (1786, 1915)*	1694 (1657, 1731)	< 0.001	1821 (1759, 1883)*	1653 (1609, 1698)	< 0.001
Food energy (kcal)	1794 (1733, 1855)*	1637 (1602, 1672)	< 0.001	1765 (1706, 1824)*	1603 (1561, 1646)	< 0.001
Protein	17.7 (17.3, 18.2)*	17.1 (16.8, 17.3)	0.001	17.7 (17.2, 18.1)*	17.2 (16.9, 17.5)	0.002
Fat	36.7 (35.9, 37.4)*	34.0 (33.6, 34.5)	< 0.001	36.4 (35.7, 37.1)*	34.0 (33.5, 34.6)	< 0.001
Saturated fatty acids	12.1 (11.7, 12.5)	12.0 (11.7, 12.2)	0.518	11.9 (11.5, 12.3)	12.1 (11.7, 12.4)	0.148
cis-Monounsaturated fatty acids	14.1 (13.7, 14.4)*	12.4 (12.2, 12.6)	< 0.001	14.0 (13.7, 14.4)*	12.4 (12.2, 12.6)	< 0.001
cis *n*-6 fatty acids	5.9 (5.7, 6.1)*	5.1 (5.0, 5.3)	< 0.001	5.9 (5.7, 6.1) *	5.1 (5.0, 5.2)	< 0.001
cis *n*-3 fatty acids	1.0 (1.0, 1.1)*	1.0 (0.9, 1.0)	0.002	1.0 (1.0, 1.1)*	1.0 (0.9, 1.0)	< 0.001
*Trans* fatty acids	0.4 (0.4, 0.5)*	0.5 (0.5, 0.5)	< 0.001	0.4 (0.4, 0.5)*	0.5 (0.5, 0.6)	< 0.001
Carbohydrate	45.2 (44.3, 46.0)*	48.6 (48.1, 49.0)	< 0.001	45.6 (44.8, 46.4)*	48.5 (48.0, 49.1)	< 0.001
Total sugars	17.9 (17.1, 18.6)	18.2 (17.8, 18.7)	0.283	17.6 (16.9, 18.4)	17.6 (17.1, 18.1)	0.838
Starch	26.1 (25.3, 26.8)*	28.6 (28.4, 29.3)	< 0.001	26.6 (25.9, 27.3)*	29.4 (28.9, 29.9)	< 0.001
Non-milk extrinsic sugars	7.6 (7.1, 8.2)*	9.6 (9.2, 10.0)	< 0.001	7.6 (7.0, 8.1)*	9.2 (8.7, 9.6)	< 0.001
Intrinsic milk sugars and starch	36.1 (35.3, 36.8)*	37.3 (36.9, 37.8)	< 0.001	36.5 (35.7, 37.2)*	37.7 (37.2, 38.3)	< 0.001
Intrinsic milk sugars	8.8 (8.3, 9.2)*	7.2 (6.9, 7.4)	< 0.001	8.7 (8.2, 9.1)*	7.0 (6.7, 7.3)	< 0.001
Non-starch polysaccharides (Englyst Fibre, g)	14.6 (14.1, 15.1)*	12.1 (11.9, 12.4)	< 0.001	14.0 (13.5, 14.5)*	11.9 (11.6, 12.2)	< 0.001
Alcohol (% total energy)	0.9 (0.7, 1.2)	1.0 (0.9, 1.2)	0.405	0.8 (0.6, 1.1)	0.9 (0.7, 1.0)	0.734

Micronutrients^b^						
Vitamin A (retinol equivalents) (μg)	638.7 (587.2, 695.0)	603.1 (574.7, 632.9)	0.124	598.5 (551.5, 649.6)	565.2 (533.2, 599.3)	0.069
Vitamin D (μg)	2.2 (2.1, 2.4)	2.2 (2.1, 2.3)	0.824	2.3 (2.2, 2.5)	2.2 (2.1, 2.3)	0.057
Vitamin E (mg)	10.7 (10.3, 11.1)*	8.6 (8.4, 8.8)	< 0.001	10.5 (10.1, 10.9)*	8.3 (8.2, 8.6)	< 0.001
Thiamin (mg)	1.4 (1.3, 1.4)*	1.3 (1.2, 1.3)	< 0.001	1.4 (1.3, 1.4)*	1.3 (1.2, 1.3)	< 0.001
Riboflavin (mg)	1.3 (1.3, 1.4)*	1.3 (1.2, 1.3)	0.047	1.3 (1.3, 1.4)*	1.3 (1.2, 1.3)	0.002
Niacin equivalent (mg)	32.5 (31.5, 33.5)	32.5 (31.9, 33.0)	0.983	32.9 (31.9, 33.9)	32.5 (31.8, 33.2)	0.277
Vitamin B6 (mg)	2.7 (2.6, 2.8)*	2.8 (2.7, 2.8)	0.028	1.7 (1.7, 1.8)	1.8 (1.7, 1.8)	0.152
Vitamin B12 (μg)	3.9 (3.8, 4.2)	4.1 (4.0, 4.3)	0.105	4.0 (3.6, 4.2)	4.0 (3.8, 4.2)	0.971
Folate (μg)	221.9 (213.1, 231.1)*	208.9 (204.1, 213.8)	0.001	218.8 (210.3, 227.6)*	202.5 (196.8, 208.2)	< 0.001
Pantothenic acid (mg)	5.1 (4.9, 5.2)*	4.9 (4.9, 5.0)	0.047	5.2 (5.0, 5.3)*	4.9 (4.8, 5.0)	< 0.001
Biotin (μg)	36.4 (34.9, 37.9)*	28.0 (27.3, 28.7)	< 0.001	35.6 (34.2, 37.1)*	27.0 (26.2, 27.8)	< 0.001
Vitamin C (mg)	83.9 (77.6, 90.8)*	63.2 (60.4, 66.1)	< 0.001	59.7 (56.5, 63.0)*	78.9 (73.1, 85.1)	< 0.001
Sodium (mg)	1614.8 (1562.1, 1668.9)*	1790.8 (1757.3, 1825.0)	< 0.001	1621.9 (1570.8, 1674.7)*	1819.1 (1778.1, 1861.1)	< 0.001
Potassium (mg)	2689.3 (2619.0, 2762.1)*	2423.9 (2387.4, 2461.1)	< 0.001	2880.8 (2811.2, 2950.5)*	2584.0 (2533.9, 2634.0)	< 0.001
Calcium (mg)	641.2 (619.7, 663.5)	624.2 (612.1, 636.5)	0.075	629.1 (608.5, 650.3)	617.3 (602.8, 632.1)	0.141
Magnesium (mg)	278.5 (271.3, 285.9)*	227.6 (224.2, 231.1)	< 0.001	270.9 (264.2, 277.9)*	223.2 (219.2, 227.3)	< 0.001
Phosphorus (mg)	1128.3 (1103.3, 1154.8)*	1060.0 (1046.6, 1073.7)	< 0.001	1130.1 (1105.6, 1154.8)*	1063.7 (1046.1, 1079.2)	< 0.001
Iron (mg)	10.3 (10.0, 10.6)*	9.5 (9.3, 9.7)	< 0.001	10.1 (9.8, 10.4)*	9.4 (9.2, 9.6)	< 0.001
Copper (mg)	1.3 (1.2, 1.3)*	1.1 (1.0, 1.1)	< 0.001	1.2 (1.2, 1.3)*	1.0 (1.0, 1.0)	< 0.001
Zinc (mg)	8.1 (7.9, 8.4)*	7.8 (7.6, 7.9)	0.001	8.0 (7.8, 8.2)*	7.7 (7.6, 7.9)	0.001
Chloride (mg)	2690.5 (2611.2, 2772.3)*	2887.7 (2838.9, 2937.3)	< 0.001	2684.3 (2607.6, 2763.4)*	2912.4 (2852.6, 2973.4)	< 0.001
Manganese (mg)	3.4 (3.3, 3.6)*	2.7 (2.6, 2.8)	< 0.001	3.3 (3.2, 3.4)*	2.6 (2.6, 2.7)	< 0.001
Iodine (μg)	122.2 (116.2, 128.4)	123.5 (121.0, 128.1)	0.624	127.3 (121.3, 133.7)	122.7 (118.5, 127.1)	0.049
Selenium (μg)	50.0 (48.0, 52.1)*	46.2 (45.2, 47.4)	< 0.001	49.2 (48.2, 51.2)*	45.3 (44.0, 46.6)	< 0.001

All data are non-normally distributed, except total energy, food energy, fat, Saturated fatty acids, cis-Monounsaturated fatty acids, carbohydrate, starch and intrinsic milk sugars and starch. Presented values from non-normally distributed data are back log-transformed

*AKO* almond kernel only, *AKM* almond kernel only plus almond kernel in mixed nuts

*P < 0.05 showed a significant difference, *n* = 6802

^a^Survey-adjusted GLM with a linear link function and predictors: age, sex, ethnicity, socio-economic and smoking status was used for energy intake as an outcome for AKO; Survey-adjusted GLM with a linear link function and predictors: age, sex, ethnicity, socio-economic and smoking status, alcohol and energy intakes was used for other macronutrient intake outcomes for AKO; Survey-adjusted GLM with a linear link function and predictors: age, sex, ethnicity, socio-economic and smoking status, and energy intake was used for alcohol intake as an outcome for AKO. The same statistical analysis was conducted for AKM but region of residency was also included into predictors. Region of residence was not significantly correlated with AKO consumption; thus it was not included as a predictor for analysis related to AKO

^b^Survey-adjusted GLM with a linear link function and predictors: age, sex, ethnicity, socio-economic and smoking status, alcohol and energy intakes was used for AKO. The same statistical analysis was conducted for AKM but region of residency was also included into predictors. Region of residence was not significantly correlated with AKO consumption; thus it was not included as a predictor for analysis related to AKO

### Cardiovascular disease risk markers

Blood samples were not available from all participants, and anthropometric and blood pressure data were also incomplete. Sample sizes and estimated marginal mean (95% CI) values of CVD risk markers for remaining participants are shown in Table 3. BMI was significantly lower for AKO by 0.8 kg/m^2^ (*P* = 0.010) and AKM consumers by 0.6 kg/m^2^ (*P* = 0.019) compared to non-consumers. WC was significantly lower for AKO consumers by 2.1 cm (*P* = 0.007), but the difference between AKM consumers and non-consumers did not reach statistical significance. Survey-adjusted regression analysis showed that there was no dose–response relationship between almond consumption and CVD risk markers (data not shown).

**Table 3:** Cardiovascular disease risk marker values of whole almond consumers and non-consumers, in the UK adult population (≥ 19 year) based on NDNS 2008–2017

Estimated marginal mean (95% CI)						
CVD risk marker	AKO^a^			AKM^b^		
	Consumers, *n* = 317 (4.7% of total adult population)	Non-consumer, *n* = 6,495 (95.3% of total adult population)	*P* value	Consumers, *n* = 481 (7.1% of total adult population)	Non-consumer, *n* = 6321 (92.9% of total adult population)	*P* value
BMI (kg/m^2^)^c^	25.5 (24.9, 26.2)	26.3 (25.9, 26.7)	0.010*	25.8 (25.1, 26.5)	26.4 (25.9, 26.9)	0.019*
WC (cm)^d^	88.0 (86.2, 89.8)	90.1 (89.1, 91.2)	0.007*	90.1 (88.3, 91.9)	91.3 (90.0, 92.6)	0.065
SBP (mmHg)^e^	119.6 (117.3, 121.9)	121.2 (119.8, 122.6)	0.114	119.7 (117.4, 122.0)	121.3 (119.6, 123.0)	0.058
DBP (mmHg)^e^	71.3 (69.8, 73.0)	71.6 (70.6, 72.6)	0.720	71.2 (69.6, 72.8)	71.8 (70.6, 73.0)	0.316
TC (mmol/l)^f^	4.8 (4.6, 5.0)	4.8 (4.7, 4.9)	0.558	4.8 (4.6, 5.0)	4.8 (4.6, 4.9)	0.485
TAG (mmol/l)^g^	1.0 (0.9, 1.1)	1.1 (1.0, 1.2)	0.130	1.1 (1.0, 1.2)	1.1 (1.1, 1.2)	0.445
HDL-C (mmol/l)^f^	1.4 (1.4, 1.5)	1.4 (1.4, 1.4)	0.222	1.4 (1.3, 1.5)	1.4 (1.3, 1.4)	0.139
LDL-C (mmol/l)^h^	2.9 (2.7, 3.0)	2.8 (2.7, 2.9)	0.476	2.8 (2.7, 2.9)	2.8 (2.7, 3.0)	0.727
TC:HDL-C^f^	3.4 (3.2, 3.6)	3.5 (3.4, 3.6)	0.533	3.5 (3.3, 3.7)	3.5 (3.4, 3.6)	0.548
CRP (mg/l)^i^	2.2 (1.9, 2.6)	2.4 (2.2, 2.7)	0.158	2.3 (2.0, 2.7)	2.5 (2.3, 2.8)	0.146

All data are not normally distributed, thereby the presented values are back log-transformed. Due to missing data, sample sizes were as follows: AKO consumers 242^c^, 247^d^, 228^e^, 184^f^, 184^ g^, 183^ h^ and 184^i^; non-consumers 4466^c^, 4645^d^, 3784^e^, 3183^f^, 3174^ g^, 3139^ h^ and 3184^i^; AKM consumers 370^c^, 380^d^, 342^e^, 274^f^, 273^ g^, 271^ h^ and 274^i^; non-consumers 4338^c^, 4512^d^, 3670^e^, 3093^f^, 3084^ g^, 3051^ h^ and 3094^i^

*AKO* almond kernel only; *AKM* almond kernel only plus almond kernel in mixed nuts; *BMI* body mass index; *WC* waist circumference; *SBP* systolic blood pressure *DBP* diastolic blood pressure; *TC* total cholesterol; *TAG* triacylglycerol; *HDL-C* high density lipoprotein cholesterol; *LDL-C* low density lipoprotein cholesterol; *TC:HDL-C* total to HDL cholesterol ratio; *CRP* C-reactive protein

*P < 0.05 showed a significant difference, *n* = 6802

^a^Survey-adjusted GLM with a linear link function and predictors: age, sex, ethnicity, socio-economic and smoking status, alcohol and energy intakes was used. Region of residence was not significantly correlated with AKO consumption; thus it was not included as a predictor

^b^Survey-adjusted GLM with a linear link function and predictors: age, sex, ethnicity, region of residency, socio-economic and smoking status and alcohol intake was used

## Discussion

Inclusion of nuts in the diet is recommended as part of the emphasis on consuming more plant-based diets for the benefit of both human health and the environment [1, 4, 7, 22]. Almonds are the most consumed tree nut in high-income economies [5], and scientific evidence has demonstrated that consumption can lower LDL-cholesterol concentrations [22, 23], which could contribute to the prevention of coronary heart disease [45]. However, only 1.6% of the US adult population reported consuming whole and processed almonds using data collected by two 24 h dietary recalls [25]. According to 4 day food records, it is reported that 7% of a nationally representative sample of the UK population, surveyed between 2008 and 2017, consumed whole almond kernels (excluding other forms of almonds) during a 4 day period year. The NHANES and NDNS data are not directly comparable as different dietary assessment methods and timeframes were used, but it could indicate that almond consumption may be more prevalent in the UK compared to the US, especially since the NHANES estimate was not restricted to whole almond kernels.

Whole almond consumers were more likely to be female, white, non-smoking, older, and living in England. Scores of diet quality were significantly higher in almond consumers, agreeing with previous findings in the USA NHANES population [46]. These observations suggest that people who follow a healthier dietary pattern are more likely to include whole almonds. This association with better diet quality was reflected in the nutrient intake analysis: consumers had a higher intake of fiber and unsaturated fatty acids, and lower intakes of non-milk extrinsic sugars. Intakes of most micronutrients, including vitamin E, thiamin, riboflavin, folate, pantothenic acid, biotin, vitamin C, phosphorus, iron, zinc, selenium, iodine, manganese, magnesium, potassium, and copper intakes were found to be higher in consumers following adjustment for energy intake, indicating that they were likely to consume a more nutrient-dense diet in general. Sodium and chloride intakes were lower in consumers indicating reduced salt consumption compared with non-consumers, in agreement with the higher scores for diet quality. Therefore, these data support the widely recommended approach that a healthy dietary pattern will include nuts as a plant-based source of protein and micronutrients. It is important to remember that the median almond intake by UK adult consumers was just 5 g/day (equivalent to four almonds), with the 75th percentile only reaching 12 g/day. This fact, alongside data from our previous study that reported the median intake of total tree nuts to be 7 g/day in UK adults [44], shows that daily intakes are far below what is considered to be one portion (28 g) of tree nuts [44], and are, therefore, unlikely to be eaten in quantities that could cause clinically meaningful LDL-cholesterol lowering effects in the majority of consumers.

Almond consumers had slightly lower BMI and WC. Similar body composition findings were observed in the US NHANES almond consumer population, using a statistical model adjusted for age, sex, ethnicity, poverty index ratio, physical activity level, current smoking status, alcohol consumption, and total energy intake [25]. Findings from randomized controlled trials have been mixed regarding the effects of almond consumption on body composition; a recent meta-analysis that pooled data from 15 trials concluded there was no difference in BMI between almond and control interventions in healthy and at risk subjects with a range of 25–100 g/day of almonds [23]. Physical activity levels were not available from the NDNS database and thus could be a confounding factor in the differences in body composition observed in this analysis.

There were no differences in blood pressure (SBP and DBP) according to almond consumption. Our previous analysis of the 2008–2014 NDNS sample showed that tree nut consumers had on average 4.3 mmHg significantly lower SBP than non-consumers, and that with every gram increase in tree nut consumption per 1000 kcal of energy intake, SBP was 0.2 mmHg lower [44]. The limited range of whole almond intakes is likely to explain the lack of dose–response relationship with SBP (and BMI and WC) in the current study. Other observational tree nut studies have reported conflicting findings for blood pressure, with reports that tree nut consumption is associated with lower SBP but not DBP in the USA NHANES database [47], but the SUN prospective cohort study found no associations at all with blood pressure [48]. No significant effect of almond intervention (dose 25–100 g/day) on SBP was reported in a meta-analysis of randomized controlled trials, but DBP was shown to be significantly decreased [23].

Whole almond consumption was not associated with a preferential lipid profile, such as higher HDL-cholesterol and lower total cholesterol, LDL-cholesterol, TAG, or the ratio of total to HDL-cholesterol. Again, the observational evidence is inconsistent with the interventional data; pooled analysis of 18 randomized controlled trials revealed HDL-cholesterol was not affected by almond consumption but there were significant reductions in total cholesterol, TAG and LDL-cholesterol [22]. These inconsistencies between observational and interventional studies may exist, because a higher dose (RCTs administered between 25 and 100 g almonds/day) is important for measurable differences in lipid profiles.

Previous literature on human clinical trials investigating the impact of almond consumption on CRP is inconsistent. Two randomized, controlled, crossover trials in adults with elevated LDL cholesterol found that 4 week and 6 week almond consumption at a dose of 50–75 g/day and 42.5 g/day, respectively, did not significantly modify CRP [15, 49], although other studies have reported reductions in CRP after 4 week almond consumption (as a replacement of 20% total energy) in subjects with type-2 diabetes [18], and after 4 weeks in healthy adults where 10% or 20% total energy was replaced by almonds [20]. A meta-analysis of 15 studies revealed that the overall difference between almond and control interventions did not reach statistical significance [23], but the number of studies is insufficient to determine whether baseline CRP status, dose and duration of intervention are important determinants.

Median almond intake was 5 g/day in the cohorts of UK consumers studied between 2008 and 2017, but the trend of whole almond consumption fluctuated across the period. The highest consumption level occurred in 2011–2012 (median 8.3 g/day), but consumption decreased to 3.9 g/day in the most recent cohort available, 2016–2017. Since intake of almonds was low in whole almond consumers, the superior diet quality of almond consumers is likely to reflect generally healthier dietary choices and patterns as shown by the higher diet quality scores observed in almond consumers versus non-consumers. If consumed in larger quantities by more individuals, whole almonds have the potential to directly improve the nutrient profile of the diet. Whole almonds are predominantly consumed as snacks and given that snacks account for 20–25% of estimated energy requirement in adults [50–55], snacking is a convenient food domain to target for improving diet quality. Almonds have higher unsaturated fat, fiber, magnesium, vitamin E and phenolics compared to typically consumed snacks in the UK [56, 57], and have been shown to improve other markers of cardiovascular health such as endothelial function (a measure of vascular health) [56] in addition to blood lipid profiles [15], compared to typically consumed snacks. However, when encouraging almonds as a snack replacement to improve health we need to be mindful of potential barriers, such as affordability amongst low income groups and market accessibility, as well as the need for increased global production of almonds to meet greater demands, which would require careful consideration of long-term environmental sustainability in terms of cropland and water.

A strength of this study is that the diet data was generated from 4 day estimated diet diaries, which are considered to be more accurate relative to 24 h dietary recalls. On the other hand, the 4 day estimated diet diary might not record almond intake that occurred on other days, leading to underreporting and misclassification of non-consumers. A further strength of the study is that it is based on a large database that is considered to be nationally representative of the UK population. Furthermore, the survey is designed to facilitate representation of dietary intakes across all days of the week, avoiding potential bias arising from differences between week-days and weekend-days [58]. However, it must be noted that a weakness of the study was that the UK NDNS database does not provide data on the proportions of almonds within mixed nuts products. Despite this, our estimates of almond proportions in these products via systematic market sampling mitigates the risk of underestimating almond consumption.

## Conclusion

UK almond consumers are characterized by overall healthier dietary patterns, which are likely to have been an important determinant of the more favorable markers of body composition observed in this group. It is unlikely that almond consumption independently determined lower adiposity in this population since intakes were very low. Encouraging snacking on nuts, including almonds, to replace snack foods high in saturated fatty acids, refined starches and free sugars may contribute to the sum effect of a healthy dietary pattern on reduced risk of cardiovascular diseases.

## Electronic supplementary material

Below is the link to the electronic supplementary material.

Supplementary file1 (PDF 103 kb)

## Abbreviations

AKM: Almond kernel only plus almond kernel in mixed nuts
AKO: Almond kernel only
BMI: Body mass index
COMA: Committee on Medical Aspects of Food Policy
CRP: C-reactive protein
CVD: Cardiovascular disease
DBP: Diastolic blood pressure
GLM: Generalized linear model
HDL-C: High-density lipoprotein
HDS: Healthy diet score
IQR: Interquartile range
LDL-C: Low-density lipoprotein
MDS: Mediterranean Diet Score
MUFA: Monounsaturated fatty acids
NDNS: National Diet and Nutrition Survey
NDNS-RP: National Diet and Nutrition Survey—Rolling Program
NHANES: National Health and Nutrition Examination Survey
PUFA: Polyunsaturated fatty acids
SBP: Systolic blood pressure
SD: Standard deviation
SFA: Saturated fats
TAG: Triglycerides
TC: Total cholesterol
WC: Waist circumference
