# Supplementary material for: Whole almond consumption is associated with better diet quality and cardiovascular disease risk factors in the UK adult population: National Diet and Nutrition Survey (NDNS) 2008–2017
Source: Eur J Nutr. 2020 May 16;60(2):643–54. doi: 10.1007/s00394-020-02270-9 (PMC7900023; doi:10.1007/s00394-020-02270-9)
Supplement: Supplementary file 1 — Supplementary file1 (PDF 103 kb) [file 394_2020_2270_MOESM1_ESM.pdf]

Whole almond consumption is associated with better diet quality and intermediary cardiovascular disease risk factors in the UK adult population: National Diet and Nutrition Survey (NDNS) 2008-2017

Vita Dikariyanto*, Sarah E. E. Berry, Lucy Francis, Leanne Smith, Wendy L. Hall*
Diet and Cardiometabolic Health Research Group, Department of Nutritional Sciences, Faculty of Life Sciences and Medicine, King's College London, London SE1 9NH, UK.
***Correspondence: vita.dikariyanto@kcl.ac.uk, wendy.hall@kcl.ac.uk; Tel.: +44 020 78484301/+44 020 78484197

Supplement

Appendix A: Market analysis of almond snacks

Table 1. Whole almond kernel portion in mixed nut or mixed nut and fruit products in the UK supermarkets

Type	Supermarket name	Total weight of the product	Total weight of whole almond kernels	% of whole almond kernels in the product	Mean value of % whole almond kernels
Mixed roasted unsalted nuts	Waitrose	50.1	9.7	19.4	28.5
	M&S	173.9	41.7	24.0	
	Co-op	30.5	7.4	24.3	
	Sainsbury's	203.0	48.8	24.0	
	Tesco	201.0	56.6	28.2	
	Asda	201.0	102.2	50.8	
	Morrisons	224.9	80.2	35.7	
	Lidl	200.7	42.8	21.3	
Mixed roasted salted nuts	Waitrose	175.1	54.4	31.1	30.5
	M&S	177.0	46.0	26.0	
	Sainsbury's	201.2	59.3	29.5	
	Tesco	203.1	72.3	35.6	
Mixed nuts and fruits	Sainsbury's, Product 1	201.6	13.7	6.8	18.0
	Sainsbury's, Product 2	200.0	25.3	12.7	
	Tesco	251.5	25.4	10.1	
	Asda, Product 1	250.0	45.7	18.3	
	Asda, Product 2	200.0	62.6	31.3	
	Morrisons	200.3	35.3	17.6	
	Lidl	200.3	58.3	29.1	

Appendix B: Diet quality scores

Table 1. Original and modified Mediterranean Diet Score (MDS) and its scoring system [30]

Food group		Scoring*	
Original MDS	Modified MDS		
Cereals	Cereals	>median	1 (else: 0)
Vegetables	Vegetables	>median	1 (else: 0)
**Fruits and Nuts**	**Fruits**	>median	1 (else: 0)
Legumes	Legumes	>median	1 (else: 0)
Fish	Fish	>median	1 (else: 0)
Meat	Meat	<median	1 (else: 0)
Dairy products	Dairy products	<median	1 (else: 0)
Ratio of unsaturated to saturated fats	Ratio of unsaturated to saturated fats	>median	1 (else: 0)
Alcohol	Alcohol	10-50 g/d for men	1 (else: 0)
		5-25 g/d for women	1 (else: 0)

*Except alcohol, scoring of each food group is based on population and sex-specific median.

Table 2. Original and modified Healthy Diet Score (HDS) and the cut-off values for scoring [31]

HDS			HDS – modified based on current UK recommendations		
Index item	Cut-off values		Index item	Cut-off values	
	Score 1	Score 0		Score 1	Score 0
Saturated fatty acids (% energy intake)	0-10	>10	Saturated fatty acids (% energy intake)	0-11[a]	>11
Polyunsaturated fatty acids (% energy intake)*	6-10	<6 or >10	Polyunsaturated fatty acids (% energy intake)	6[b]-10	<6 or >10
Protein (% energy intake)	10-15	<10 or >15	Protein (% energy intake)	9[c]-15	<9 or >15
Total carbohydrate (% energy intake)*	50-70	<50 or >70	Total carbohydrate (% energy intake)	50[d]-70	<50 or >70
Dietary fiber (g)*	18-32	<18 or >32	Dietary fiber (g)	18[d]-32	<18 or >32
Fruits and vegetables (g)	≥400	<400	Fruits and vegetables (g)	≥400[e]	<400
Pulses and nuts (g)*	≥30	<30	Pulses (g)	≥30	<30
Total non-milk extrinsic sugar (% total energy intake)*	0-10	>10	Total non-milk extrinsic sugar (% total energy intake)	0-5[d]	>5
Cholesterol (mg)*	0-245	>245	Trans-fatty acids (% energy intake)	≤2[e]	>2
Fish (g)*	≥32	<32	Fish (g)	≥40[e]	<40

Red meat and meat processed products (g)*	≤90	>90	Oily fish (g)	≥20[e]	<20
Calcium (mg)*	≥700	<700	Red meat and meat processed products (g)	≤70[i]	>70
			Calcium (mg)	≥700[c]	<700
			Sodium (mg)	≤2400[c]	>2400

* Items based on advice on healthy eating as recommended by the UK Committee on Medical Aspects of Food Policy (COMA) [31]

a Based on NDNS Results from Years 7 and 8 (combined) of the Rolling Program (2014/2015 – 2015/2016) [29], UK Government Dietary Recommendations by Public Health England (2016) [32], Cardiovascular Disease Outcomes Strategy by UK Department of Health (2013) [33] and Draft report: Saturated fats and health by Scientific Advisory Committee on Nutrition (SACN) 2018 [35]

b Based on UK Government Dietary Recommendations by Public Health England (2016) [32]

c Based on UK Government Dietary Recommendations by Public Health England (2016) [32]

d Based on UK Government Dietary Recommendations by Public Health England (2016) [32] and Scientific Advisory Committee on Nutrition (SACN) Report 2015 on Carbohydrates and Health [36]

e Based on NDNS Results from Years 7 and 8 (combined) of the Rolling Program (2014/2015 – 2015/2016) [29] and The Eatwell Guide by Public Health England (2016) [34]
